# Widely Tunable Dual‐Wavelength Spacing via Temperature Control in A Single Self‐Assembled Nanowire‐Well Chip With Stress Modification

**DOI:** 10.1002/nap2.70331

**Published:** 2026-10-16

**Authors:** Yuhong Wang, Yiwei Zhang, Zihan Jiang, Jian Wu, Chunqing Gao

**Affiliations:** ^1^ School of Optics and Photonics Beijing Institute of Technology Beijing China; ^2^ Key Laboratory of Information Photonics Technology Ministry of Industry and Information Technology Beijing China; ^3^ School of Physics Beihang University Beijing China

**Keywords:** nanowire‐well structure, single chip, thermal control, widely tunable dual‐wavelength difference

## Abstract

Dual‐wavelength single‐chip laser sources not only provide multiparameter measurement, but also provide a promising approach for terahertz (THz) signal generation. THz emission relies on the wavelength (or frequency) difference between dual lasing. Wide tunability of the dual‐wavelength difference, therefore, enables widely tunable THz signals for more flexible applications. In this paper, we propose a simple approach and mechanism for achieving dual‐wavelength emission with a widely tunable center‐wavelength spacing on a single chip via unified temperature control. It is based on an InGaAs self‐assembled nanowire‐quantum well composite structure with laterally periodic nanowire‐well and pure‐well parts contributing to dual‐wavelength emission. Due to distinct stress conditions in the two parts, their emission peaks exhibit markedly different temperature‐dependencies. As the temperature increases from 289 K to 321 K, the emission peak of the nanowire‐well part only varies from ∼930.6 to ∼931.8 nm (Δ ≈ 1.2 nm), while that of the pure‐well part shows a pronounced redshift from ∼963.8 to ∼985.7 nm (Δ ≈ 21.9 nm). Consequently, the dual‐wavelength spacing expands from ∼33.2 to ∼53.9 nm (Δ ≈ 20.7 nm), corresponding to a tuning rate of ∼0.65 nm/K. This research holds significant importance for enhancing the dynamic‐adaptability and compactness of dual‐wavelength semiconductor lasers for advanced applications.

## Graphical abstract

A novel and simple solution for achieving dual‐wavelength emission with widely tunable dual‐wavelength spacing on a single InGaAs self‐assembled nanowire‐quantum well composite structure chip based on thermal modulation is proposed. This research holds significant importance for enhancing the dynamic‐adaptability and compactness of dual‐wavelength semiconductor lasers for advanced applications.

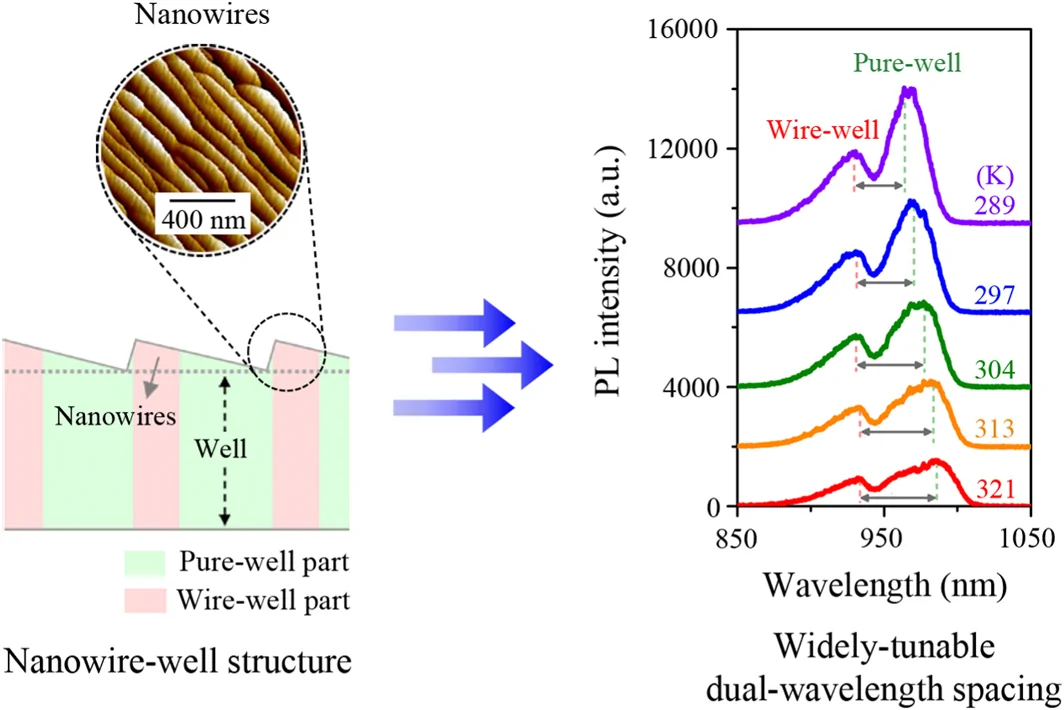

## 1 Introduction

Dual‐wavelength laser sources, capable of simultaneous or alternating emission at two distinct wavelengths, are key components in applications such as interferometry [1, 2], terahertz (THz) signal generation [3, 4, 5, 6], and microwave generation [7, 8, 9, 10]. To achieve dual‐wavelength output, several different approaches and laser systems have been proposed, such as solid‐state lasers, fiber lasers, and semiconductor lasers [11, 12, 13, 14]. Among them, semiconductor single‐chip lasers with their potential for compact size, low cost, and integrability with other photonic components have garnered increased interest from researchers. Furthermore, for dual‐wavelength single‐chip laser sources, if the wavelength or frequency difference (dual‐wavelength spacing) also possesses wide‐range tunability, it enables dynamic adaptability and multiparameter measurement [15], which will further and more significantly enhance their application value in advanced sensing, communication, and measurement systems. For instance, THz signal generation relies on the frequency difference between the two wavelengths, and achieving wide‐range tunability of this frequency difference is key to generating THz signals with widely tunable frequencies. This facilitates the development of compact, widely tunable signal sources that meet the growing demand for miniaturization and portability in optoelectronic devices, while also providing a flexible, broadband, high‐performance carrier for the advancement of communication and radar systems.

In tunable semiconductor lasers, temperature tuning offers advantages such as high wavelength stability, strong system integration, and simple control [16, 17]. These merits are especially notable when compared to alternative tuning methods. Mechanical tuning, for instance, often suffers from drawbacks including susceptibility to wear in moving parts, vibration sensitivity, as well as poor reliability and long‐term stability. Similarly, current tuning typically faces constraints such as a narrow tuning range, high losses, and poor wavelength stability [18, 19, 20]. Temperature tuning has thus become one of the most commonly used methods for direct tuning in tunable semiconductor lasers primarily achieved by altering the energy band structures of the gain media and the lengths of the optical resonators [21, 22, 23]. However, for dual‐wavelength tunable lasers, changing the temperature typically causes both wavelengths from the same gain chip to shift in the same direction. Moreover, due to their similar materials and physical structures, their temperature tuning coefficients are usually very close, resulting in a highly consistent response to temperature changes [24, 25]. Consequently, the tuning range of the wavelength difference is very limited under temperature‐based tuning [26, 27, 28, 29, 30, 31].

To address the limited tuning range of the wavelength difference under thermal control, common strategies include using two separate temperature‐control units to independently regulate two lasers or locking one wavelength while tuning the other via temperature variation [32, 33]. Although these approaches help to break the synchronous temperature‐dependent drift of the dual lasing wavelengths, the complex experimental setups required increase both the fabrication cost and implementation difficulty of such laser systems.

Suppressing the lattice thermal vibrations of the gain material through multiaxial compressive stresses is an effective approach to reduce the thermal redshift of the emission wavelength in the gain structure [34, 35, 36]. In the same chip structure, by applying different degrees of compressive stresses to two types of emission parts, the two emission peaks can exhibit distinct responses to temperature. This will serve as an effective mechanism and method for achieving dual‐wavelength emission with a widely tunable wavelength difference in a single‐chip structure based on thermal regulation. This approach is also complementary to existing multiwavelength lasers based on arrayed waveguide grating (AWG)/distributed Bragg reflector (DBR) filtering combined with optical feedback [37, 38, 39, 40], while enabling continuous tuning of the wavelength. Guided by this concept, we utilized the multiatomic step effect induced by a slightly misoriented substrate to epitaxially grow an InGaAs self‐assembled nanowire‐quantum well composite structure. Due to the uniqueness of the growth mechanism, the obtained on‐well nanowires exhibit a cross‐sectional distribution similar to an obtuse triangle. The significant height variation results in limited effective coverage of the nanowires over the quantum well, thereby forming two laterally periodic parts in the gain part, namely the nanowire‐well part and the pure‐well part, leading to dual‐wavelength emission. In the nanowire‐well part, the nanowires are subjected to multiaxial compressive stresses, exhibiting strong thermal stability and making the central wavelength of this part highly insensitive to temperature variations. In contrast, in the pure‐well part, the quantum well is under biaxial compressive stresses, and its lattice undergoes intense thermal vibrations as temperature increases, resulting in a significant thermal redshift of the central wavelength in this part. Therefore, on the same single chip and under unified temperature control, the two emission wavelengths demonstrate different temperature response characteristics, enabling a very wide wavelength difference tuning range based on unified temperature modulation. This holds significant importance for enhancing the tuning capability, dynamic adaptability, multiparameter measurement, performance optimization, and compactness of dual‐wavelength semiconductor lasers.

## 2 Material Structure and Characterizations

### 2.1 Material Structure

The GaAs(001) substrate with a slightly misoriented angle is utilized in our experiment, ensuring the spontaneous formation of the initial multiatomic steps on the subsequently deposited 200 nm‐thick GaAs layer [41, 42, 43]. Based on these initial GaAs multiatomic steps, InGaAs nanowires are formed on the InGaAs quantum well surface through a self‐assembled process in the active part, promoting the fabrication of the InGaAs nanowire‐well composite active structure. Figure 1a illustrates a schematic of the epitaxial kernel structure of this nanowire‐well composite sample, mainly featuring horizontally arranged nanowires grown on the quantum well surface in the active part. GaAsP serves as the barrier material. Between the InGaAs active layer and GaAsP barrier layers are GaAs strain‐buffer layers, which are important in the structure fabrication since they will provide strain buffering between the initial In_0.17_Ga_0.83_As active layers and GaAs_0.92_P_0.08_ barrier layers with a larger lattice difference, and hence, high‐quality material structures can be obtained. The AlGaAs layers act as both waveguide layers and the main media for absorbing the pump light.

**FIGURE 1:**
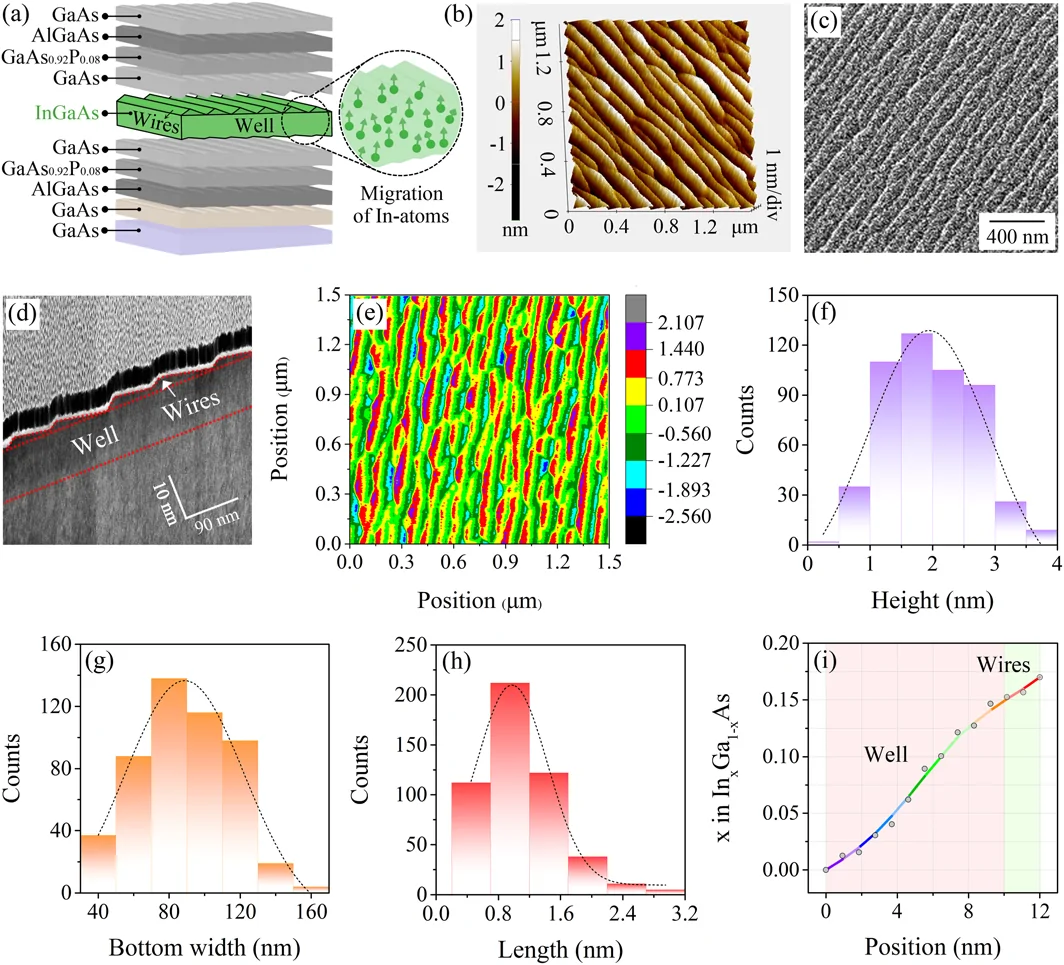
(a) Schematic representation of the nanowire‐well structure and In‐atom migration within the active layer. Representative (b) 3D, (c) top, and (d) side views of on‐well nanowires based on AFM, SEM, and cross‐sectional TEM characterizations, respectively. (e) Point‐by‐point mapping of nanowires measured over a random area of 1.5 × 1.5 μm^2^. Statistical histograms (bars) and corresponding Gaussian fits (black dashed lines) of the (f) height (purple), (g) bottom width (orange), and (h) length (red) distributions of nanowires obtained from 500 randomly selected wires. (i) In‐content profile in the nanowire‐well active layer along the material growth direction measured by XPS.

The initial indium (In)‐content of *x* = 0.17 in the In_x_Ga_1‐x_As and well‐thickness of 10 nm are designed in the active layer. This design not only supports high‐power output at the wavelength range of 900–1000 nm for many advanced applications [44, 45], but also facilitates the occurrence of the In‐segregation effect. This, in turn, promotes the spontaneous migration of In atoms along the material growth direction in the InGaAs material [46, 47], as schematically shown on the right side of Figure 1a, ensuring that the lattice constant of the on‐well nanowires exceeds that of the quantum well itself. As a result, the nanowires can be subjected to multiaxial compressive stresses, which play a crucial role in stabilizing the emission wavelength of the wire‐well part against temperature variations, thereby enabling wide thermal tuning of the dual‐wavelength spacing within a single chip. A detailed analysis of this mechanism follows in the subsequent section.

### 2.2 Sample Fabrication

The trimethylindium (TMIn), trimethylgallium (TMGa), arsine (AsH_3_), phosphine (PH_3_), and trimethylaluminium (TMAl) serve as source materials, with high‐purity hydrogen (H_2_) as the carrier gas, for the nanowire‐well sample fabrication within the metal‐organic chemical vapor deposition (MOCVD, aix200/4) system. The temperatures of the TMGa and TMIn source bottles are set at 0°C and 17°C, respectively. The growth is conducted under a total flow rate of ∼13 slm, a reaction chamber pressure of 100 mbar, and a V/III ratio of approximately 50. The growth procedure is as follows. Upon heating, the reaction chamber to 300°C, an AsH_3_ flow of 100 sccm is introduced. The temperature is then raised to 700°C for a 10‐min thermal deoxygenation step. Subsequently, the temperature is lowered to 650°C for epitaxial growth at a rate of ∼1 μm/h. After the deposition of the InGaAs active layer, the metal‐organic compound sources are switched off. The compound chamber pressure is gradually increased to 1000 mbar while maintaining continuous flows of H_2_ and AsH_3_ with the AsH_3_ flow being ramped down to 20 sccm. For the cooling phase, the AsH_3_ supply is shut off when the temperature reaches 250°C. At 180°C, the H_2_ carrier gas is replaced with nitrogen (N_2_) gas. Finally, the reaction chamber is purged after cooling to 100°C completing the growth process and allowing for sample extraction.

### 2.3 Structural Characterizations

The morphological and distribution characteristics of the nanowires are described using atomic force microscope (AFM, XE100), scanning electron microscope (SEM, Zeiss gemini300), and cross‐sectional transmission electron microscope (TEM, JEM‐2100F) techniques, and the results are presented in Figure 1b–d. Here, a dedicated sample is grown and terminated immediately after the formation of the complete nanowire‐well active layer for this analysis. It is observed that the nanowires are horizontally, well‐orderly, and periodically arranged on the quantum well surface. Owing to the multiatomic‐step‐guided growth mechanism, the cross section of the nanowire exhibits an obtuse‐triangular shape with a wide vertex angle and a narrow base angle, as displayed in the TEM image of Figure 1d. For clarity, the outlines of the nanowires and the quantum well are marked with red dotted lines. Here, the white‐gray and black layers on the nanowire surface are coatings that are formed during the fabrication process of the nanowire‐well structure sample for TEM observation.

Since this composite structure is formed through a self‐assembly process, different nanowires exhibit certain variations in height, bottom width, and length. Additionally, a single nanowire also shows height and bottom width fluctuations, as illustrated in the point‐by‐point mapping image over a randomly selected 1.5 × 1.5 μm^2^ area in Figure 1e. Moreover, size statistics are summarized in Figure 1f–h. Analysis of 500 randomly selected positions for nanowire dimensions in this 1.5 × 1.5 μm^2^ area reveals that the height, bottom width, and length can be well fitted by Gaussian functions centered at 2 nm, 90 nm, and 1 μm, respectively, as indicated by the black dashed lines in the figures. As expected from the In‐segregation effect, the In‐content increases continuously along the material growth direction within the active layer. Specifically, in the well, the In‐content increases from *x* = 0 to *x* = 0.15 in the In_x_Ga_1‐x_As material, whereas in the nanowire layer, it further increases from *x* = 0.15 to *x* = 0.17, as shown in Figure 1i, which is obtained through X‐ray photoelectron spectroscopy (XPS, PHI Quantera II) measurement.

The optical performance of the nanowire‐well structure is evaluated by measuring photoluminescence (PL) spectra. The experimental measurement result of the PL spectrum is presented in Figure 2a using a dark‐gray curve. The red and green shadows illustrate the bi‐Gaussian fitting result of the PL spectrum. Clearly, the PL spectrum exhibits dual emission peaks, with their positions located at ∼931 nm (red shadow) and ∼970 nm (green shadow), respectively. Based on our previous research and analysis, we conclude that these two peaks are attributed to the contributions of the wire‐well part and the pure‐well part [48, 49, 50, 51], as indicated by “Wire‐well” and “Pure‐well” in Figure 2a. According to the TEM characterization results, the cross‐section of the nanowires presents an obtuse‐angled triangle shape. The significant height variation results in limited effective coverage of the nanowires over the quantum well. The parts that are not effectively covered by the nanowires only function as a pure quantum well. Therefore, the entire nanowire‐well structure can be divided into two periodically distributed parts: the wire‐well part, where both the nanowires and the quantum well contribute; and the pure‐well part, where only the quantum well functions, as schematically illustrated in Figure 2b. Furthermore, due to the quantum‐size effect, the nanowires exhibit a larger band gap than the quantum well for the same material composition [52]. Consequently, it further widens the unified band gap of the wire‐well part relative to the pure‐well part due to the coupling between the nanowires and the quantum well. Therefore, the shorter‐wavelength emission originates from the wire‐well part, whereas the longer‐wavelength emission comes from the pure‐well part, as observed in the PL spectrum of Figure 2a.

**FIGURE 2:**
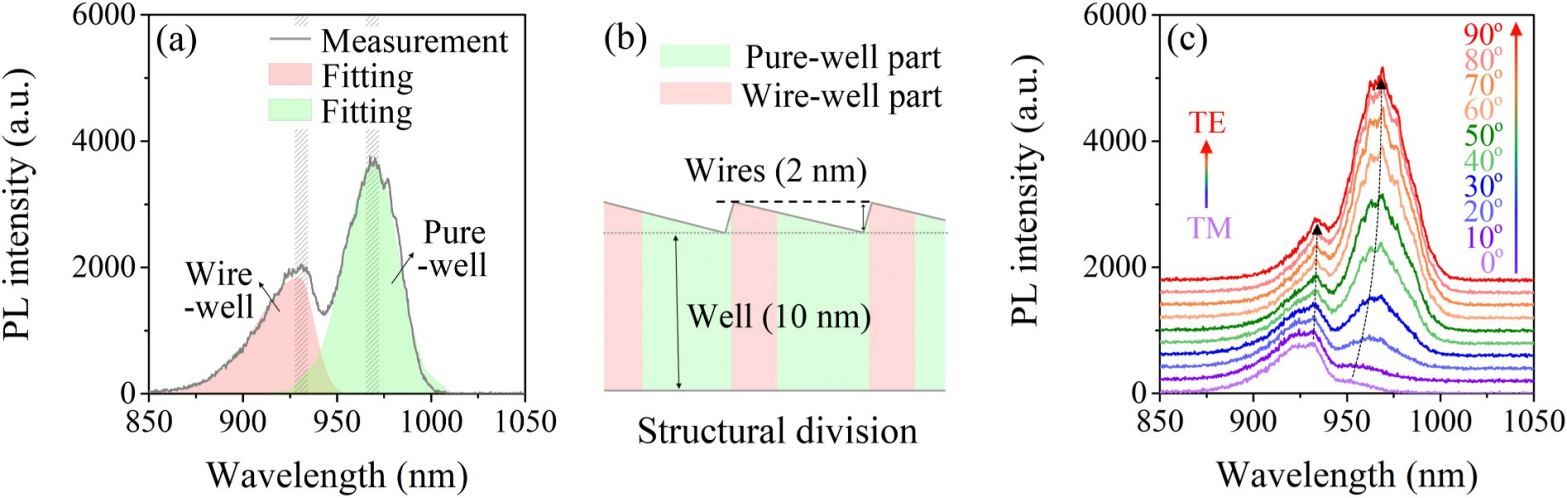
(a) PL spectrum analysis with the experimental data and the bi‐Gaussian fittings. (b) Structural division of the pure‐well part and the wire‐well part within the entire nanowire‐well structure. (c) Polarization‐dependent PL spectra from TM to TE mode.

To exclude the possibility that the short‐wavelength peak originates from a sub‐level of the pure‐well part, the polarized PL signals are measured by setting the polarizer angle at *θ* = 0°, 10°, 20°, 30°, 40°, 50°, 60°, 70°, 80°, and 90°, respectively, and the corresponding spectra are collected, as exhibited in Figure 2c. Here, the vertical direction of the magnetic‐field vector is defined as the 0° polarizer angle, where the polarizer is set in the vertical (material growth) direction, corresponding to the transverse magnetic (TM)‐polarized situation. Similarly, the vertical direction of the electric‐field vector is defined as the 90° polarizer angle, where the polarizer is set in the horizontal (in‐plane) direction of the sample, corresponding to the transverse electric (TE)‐polarization case. As is visible in Figure 2c, for both the pure‐well part and the wire‐well part, the TE‐mode emission peaks are redshifted relative to the TM‐mode peaks. This is primarily because the combined stresses applied to both types of parts are compressive stresses. Additionally, as the polarization changes from TM mode to TE mode, the redshift magnitude of the center wavelength in the pure‐well part is larger than that in the wire‐well part due to the stronger combined compressive stresses applied to the pure‐well part, which has been analyzed in detail in our previous work [49].

Here, the measurements of polarized and unpolarized PL spectra are performed using the schematic setup shown in Figure 3. In this setup, an 808‐nm fiber‐coupled laser (LWIRL808‐40W‐F) serves as the pump source to perform optical pumping on the nanowire‐well sample from the top, and the pump beam is reshaped from a Gaussian to a flat‐top profile to ensure uniform carrier injection along the axial direction. To minimize thermal effects, the pump laser is operated in pulsed output (1 Hz frequency, 20 ms pulse width). Owing to the one‐dimensional nanowires exhibiting anisotropic optical properties [53], all PL signals are collected along the direction parallel to the nanowires to maximize signal strength. The linear polarizer together with two lenses (Lens_1_ and Lens_2_) is used to obtain a converged polarized light signal. When the polarizer is oriented parallel to the sample plane, TE‐polarized light signals are collected. When the polarizer is oriented perpendicular to the sample plane, TM‐polarized light signals are collected. In addition, unpolarized light signals are obtained by removing the polarizer. The sample temperature is controlled via a thermoelectric cooler (TEC). All optical signals in the experiments are measured using a fiber‐coupled spectrometer (HR4000CG‐UV‐NIR).

**FIGURE 3:**
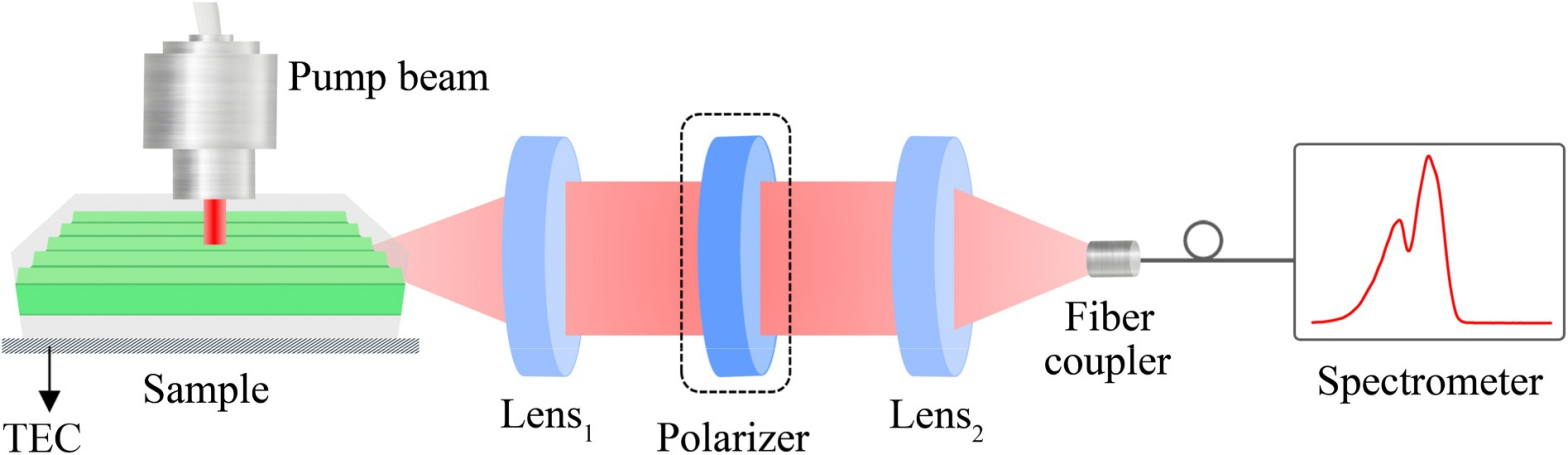
Schematic diagram of the experimental setup for unpolarized (without polarizer) or polarized PL (with polarizer) spectral measurements of a laser‐pumped edge‐emitting sample.

In addition, in the TE‐polarized PL spectrum, the peak intensity in the short‐wavelength direction is lower than that in the long‐wavelength direction. However, in the TM‐polarized PL spectrum, the peak intensity in the short‐wavelength direction is higher than that in the long‐wavelength direction. This dispels the speculation that the peak in the short‐wavelength direction is attributed to the contribution of the sub‐energy level in the pure‐well part, confirming the rationality of our previous structural division. The light emission in the short‐wavelength direction mainly originates from the wire‐well part, whereas the light emission in the long‐wavelength direction mainly comes from the pure‐well part. This structural division is also fully consistent with the theoretical modeling analysis in our previous work [48]. Additionally, the nanowire‐well structure used in this study was recently researched and developed by our group [48, 49, 50, 51, 54, 55]. The characterization results of this structure obtained here are consistent with our previous work. Although earlier studies highlight the performance advantages of this structure in areas such as depolarization and ultra‐wide gain, the present work focuses on its potential for achieving widely tunable dual‐wavelength spacing via temperature control.

This nanowire‐well composite structure is a self‐assembled structure formed by the combined effects of multiatomic steps induced by the slightly misoriented GaAs(001) substrate and In segregation. To verify the reproducibility of this structure, three batches of samples are fabricated under identical experimental conditions, and these PL spectra are measured under the same conditions. The normalized PL spectra are shown in Figure 4a, in which the essentially consistent spectral features demonstrate the good reproducibility of this composite structure.

**FIGURE 4:**
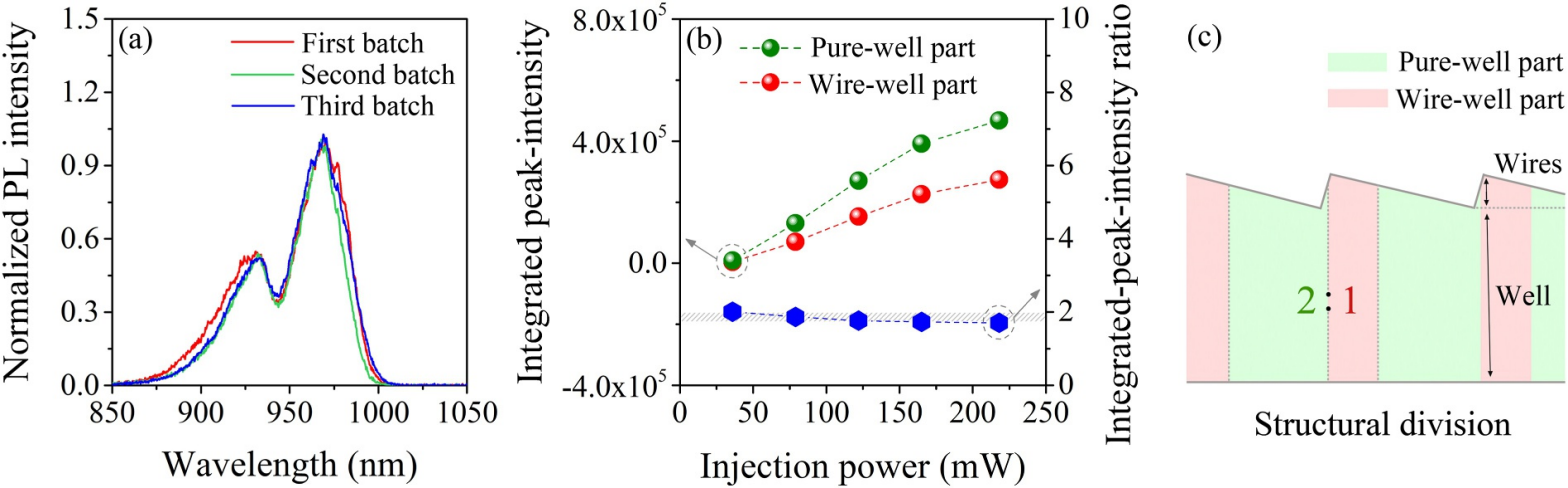
(a) Normalized PL spectra of different batches of the nanowire‐well samples fabricated and measured under the same conditions. (b) Integrated peak‐intensity of PL spectra and the corresponding integrated‐peak‐intensity ratio of these two kinds of parts (I_pure‐well_/I_wire‐well_). (c) The proportion of the pure‐well part and the wire‐well part in the entire nanowire‐well structure.

To further study the proportion of the wire‐well part and the pure‐well part in the entire nanowire‐well structure, the integrated peak‐intensities of PL spectra for the pure‐well and the wire‐well parts are derived from measurements, fitting, and calculations at various injection power levels and are plotted against injection power in Figure 4b (297 K). These two types of parts exhibit a continuous strengthening of their PL peak intensities throughout the measured injection power range. However, the integrated‐peak‐intensity ratio (I_pure‐well_/I_wire‐well_) maintains a near‐constant value of ∼2 across the entire injection power range, showing no significant fluctuation. Thus, it can be approximately concluded that the structural ratio of the pure‐well part to the wire‐well part is 2:1, as schematically exhibited in Figure 4c.

## 3 Results and Discussions

### 3.1 Independence of the PL Peak Center Wavelength and the Dual‐Peak Spacing on the Injection Power

As a nondestructive and direct optical characterization technique, PL spectroscopy serves to reveal the luminescent properties of the nanowire‐well structure, providing a direct basis for evaluating its performance in photonic devices. To investigate the injection‐dependent behavior of the emission wavelength, the PL spectra of the nanowire‐well sample are measured under injection powers ranging from 36 to 218 mW at 289 K (Figure 5a). Notably, the PL peak wavelengths for both the pure‐well and wire‐well parts show negligible dependence on the injection power, as is evident from the data in Figure 5a,b. In other words, the peak wavelengths of both parts are injection‐independent. To further verify this finding, the relationship between PL center wavelength and injection power is characterized for both structural parts across a range of temperatures (289, 297, 304, 313, and 321 K). The results are presented separately in Figure 5c (wire‐well part) and Figure 5d (pure‐well part). Clearly, under all temperature conditions, the positions of the dual PL peaks, each corresponding to one structural part, remain unaffected by changes in injection power. This injection independence also ensures that the spacing between the two peaks stays nearly constant across the injection range.

**FIGURE 5:**
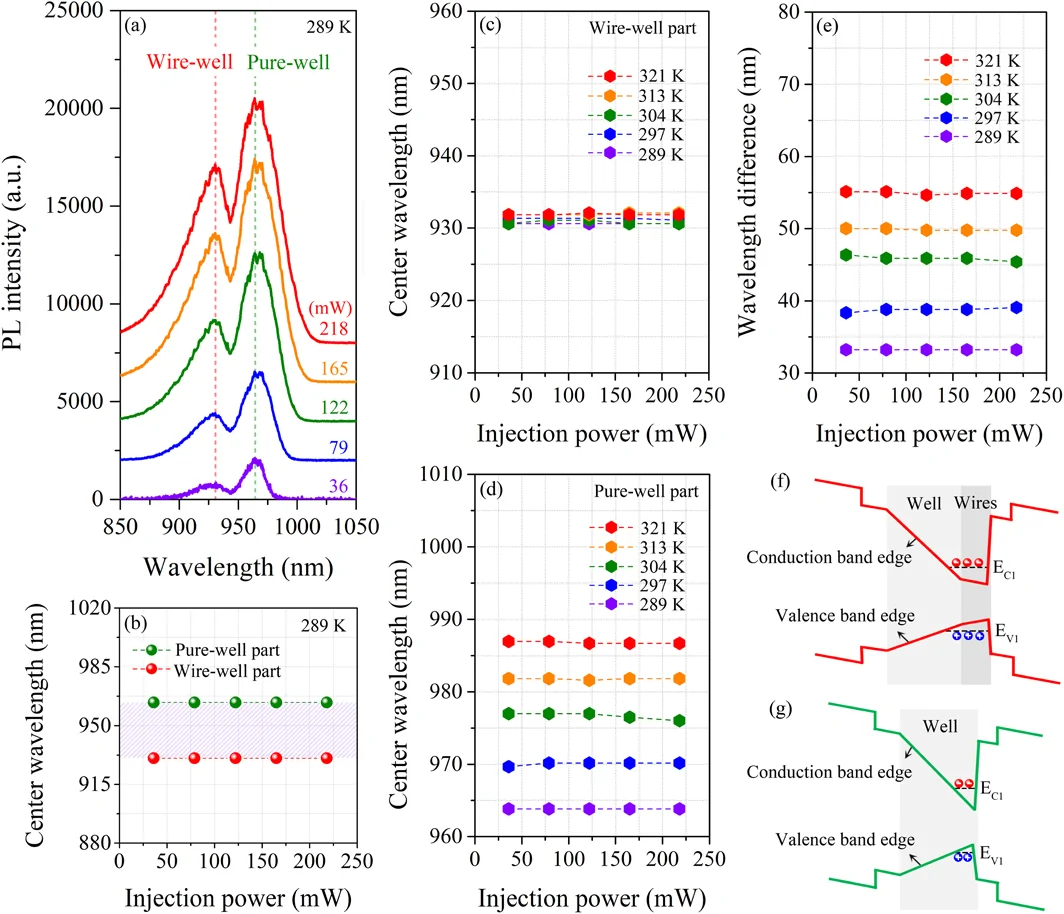
(a) PL spectra of the nanowire‐well sample measured at 289 K under injection powers ranging from 36 to 218 mW. (b) Center wavelength position of the PL spectrum as a function of injection power for both the pure‐well and wire‐well parts at 289 K. Characteristic curves of center wavelength versus injection power under different temperatures (289, 297, 304, 313, and 321 K) for (c) the wire‐well part and (d) the pure‐well part. (e) Center wavelength difference between the pure‐well and the wire‐well parts as a function of injection power. Schematic band diagrams of (f) the wire‐well part and (g) the pure‐well part.

The consistency of injection‐independent dual‐peak spacing at various temperatures is confirmed in Figure 5e. The observed injection‐independent dual‐peak wavelengths in the nanowire‐well structure can be attributed primarily to strong carrier confinement, accompanied by weakened band‐filling and quantum‐confined Stark effects. In terms of the energy band structure (Figure 5f,g), this is manifested as follows: as injection increases, carriers remain tightly confined to fixed quantized energy levels (e.g., *E*_c1_ and *E*_v1_, the first conduction and valence band levels, respectively) before radiative recombination. Here, due to the In‐segregation effect during material growth, the In‐content in the active part increases along the growth direction [46, 47], leading to a gradual band‐edge tilting within the active layer. In addition, since the quantum well and nanowires are directly coupled through the nanowire‐well interface, the nanowire‐well part forms a unified set of energy levels, as illustrated in Figure 5f.

For strained material systems, which are often expected to emit polarized light for specific applications [56, 57], evaluating their polarization properties is generally necessary. This is also implemented in our nanowire‐well active structure, which is realized in an InGaAs/GaAs/GaAsP strained material system. The TE‐ and TM‐polarized PL spectra in Figure 6a,b, measured across a range of injection powers (68–304 mW), show that the peak wavelengths for both the pure‐well and wire‐well parts remain injection‐insensitive under both polarization modes. Consequently, the dual‐peak spacing remains stable with varying injection power under polarization modes. To further validate the generality of this injection‐insensitive behavior beyond optical pumping, we also perform electrical injection measurements. The corresponding electroluminescence (EL) spectra, acquired using a continuous current source (LDC220C), are presented in Figure 6c. Clearly, the EL peak positions for both structural parts also remain stable under varying electrical injection currents. Stable dual‐wavelength spacing is also demonstrated in electrically injected samples.

**FIGURE 6:**
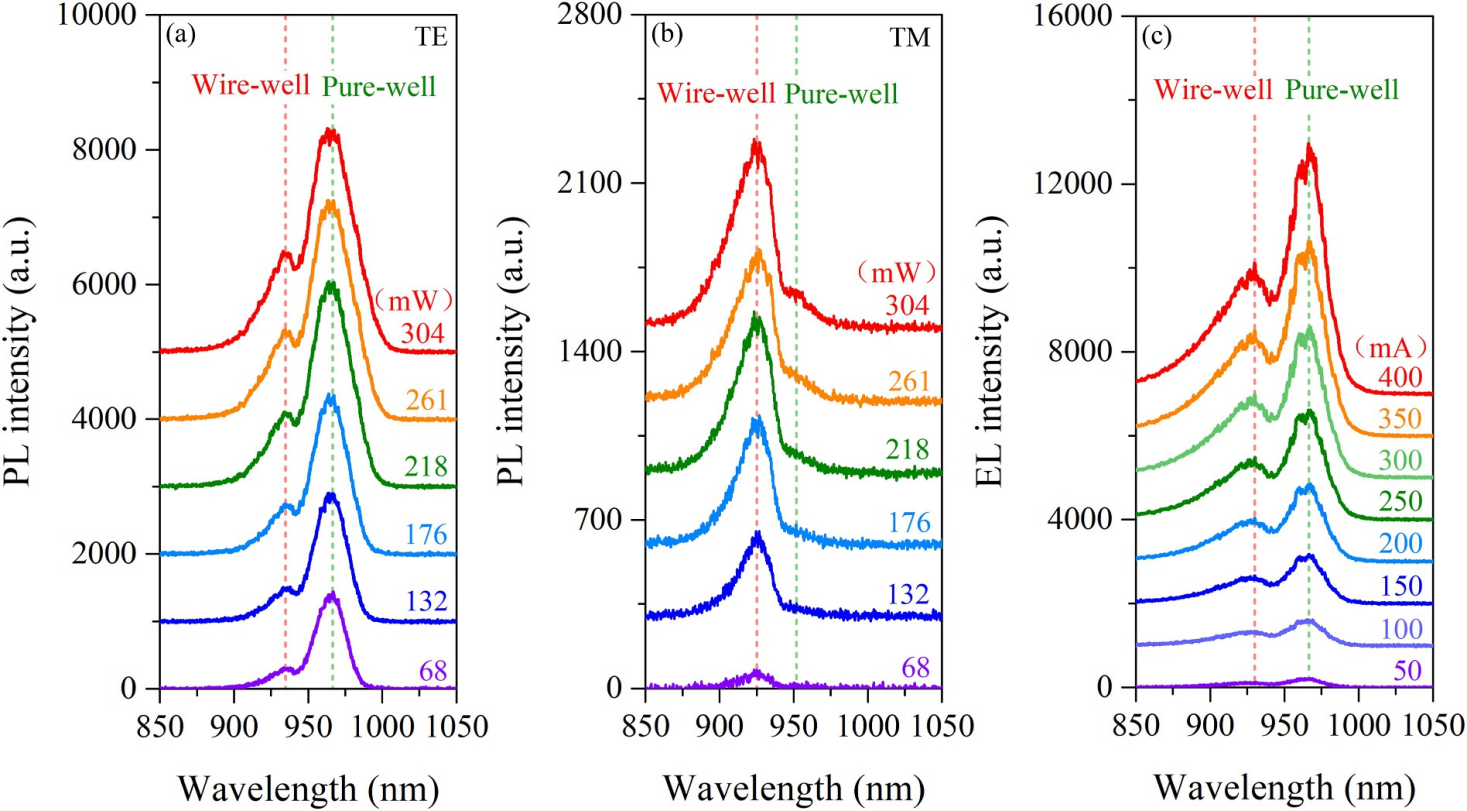
(a) TE‐ and (b) TM‐polarized PL spectra of the nanowire‐well sample measured at 297 K as a function of injection power (68–304 mW). (c) Corresponding EL spectra under 297 K across an injection current range of 50–400 mA.

### 3.2 Temperature Dependence of the PL Dual‐Peak Spacing

Near‐room‐temperature and high‐temperature environments are more aligned with the practical application requirements of most optoelectronic devices. Moreover, low‐temperature tuning necessitates additional cooling equipment, increasing system complexity and power consumption. To achieve a portable, low‐cost, wide‐range temperature‐based dual‐wavelength tuning mechanism and method, this study focuses on the room‐temperature to high‐temperature range. By measuring the PL spectra at various temperatures (289, 297, 304, 313, 321, and 330 K) under an injection power of 79 mW (Figure 7a), the distinct temperature‐dependent behaviors of the PL peak wavelengths from the pure‐well and wire‐well parts can be observed. A notable feature is that the peak wavelength of the wire‐well part remains relatively stable and only varies from ∼930.6 to ∼931.8 nm (Δ ≈ 1.2 nm), whereas that of the pure‐well part exhibits a clear red shift from ∼963.8 to ∼985.7 nm (Δ ≈ 21.9 nm) as temperature increases from 289 to 321 K (Figure 7b). The PL dual‐peak spacing expands from ∼33.2 to ∼53.9 nm (Δ ≈ 20.7 nm), corresponding to a tuning rate of approximately 0.65 nm/K at the temperature range of 289–321 K. As the temperature continues to rise (from 321 to 330 K), the wavelength in the wire‐well part remains stable, while the redshift of the wavelength in the pure‐well part becomes extremely slow due to the combined effects of a decelerating band‐gap narrowing rate, intensified carrier thermal escape, and increased nonradiative recombination. Across different injection powers (36, 79, 122, 165, and 218 mW), the temperature‐dependent wavelength shift trend remains consistent, as confirmed by the data in Figure 7c–e. This asynchronous temperature‐dependent shift of the two emission wavelengths offers an innovative mechanism for temperature‐driven tuning of the dual‐wavelength spacing within a single structure under unified temperature control. To maintain high tuning efficiency, this mechanism is not recommended at temperatures exceeding 321 K. This optical tuning capability enables controllable, wide‐range tuning of the dual‐peak spacing on a single chip, highlighting the potential of the nanowire‐well structure for achieving widely spacing‐tunable dual‐wavelength laser operation.

**FIGURE 7:**
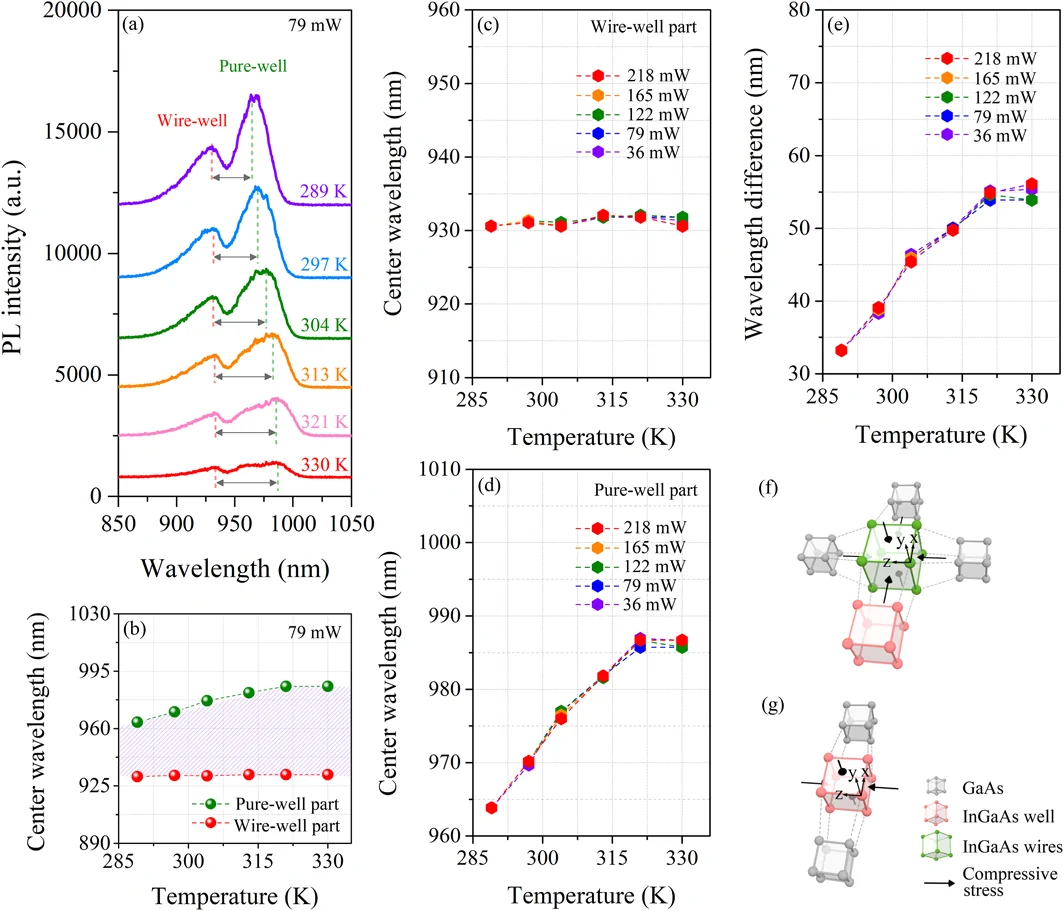
(a) PL measured spectra across temperatures ranging from 289 to 330 K at an injection power of 79 mW. (b) PL center wavelength as a function of temperature for the pure‐well and wire‐well parts under 79 mW injection. Temperature‐dependent shift of the PL peak wavelength from 289 to 330 K for (c) the wire‐well part and (d) the pure‐well part measured at injection powers of 36, 79, 122, 165, and 218 mW. (e) Dual‐peak wavelength spacing between the two parts versus temperature at different injection powers. Schematic illustration of compressive stress applied to (f) the nanowires in the wire‐well part and (g) the quantum well in the pure‐well part, represented using lattice models.

However, temperature‐sensitive, wide‐range tuning of the dual‐wavelength spacing also makes the dual‐wavelength spacing and the resulting beatnote susceptible to uncontrolled temperature fluctuations. For example, at 289 K, with initial dual wavelengths of 930.6 and 963.8 nm, a temperature change of 0.1 K leads to a beatnote change of approximately 20.8 GHz. Therefore, uncontrolled temperature fluctuations can cause undesired drift in the dual‐wavelength spacing and the beatnote, affecting the stability and precision of tuning. For practical applications, active temperature control and/or feedback locking are therefore required to ensure stable and precise wavelength tuning.

The distinct temperature responses of the PL peaks from the wire‐well and pure‐well parts originate primarily from their different stress configurations. In general, temperature‐dependent shifts in emission wavelength arise from electron‐lattice interactions and lattice thermal expansion, which alter the energy band gap [21]. The presence of multiaxial compressive stresses, however, can strongly suppress lattice thermal expansion, leading to temperature‐stable emission [34, 36]. Owing to the In‐segregation effect, the nanowires possess a higher In‐content (*x* in In_x_Ga_1‐x_As) than the quantum well in the wire‐well part (Figure 1i). This results in a larger lattice constant for the nanowires, according to Vegard's law [58]. Consequently, the nanowires experience multiaxial compressive stress: along the y‐ and *z*‐axes from the underlying quantum well; along the y‐ and *x*‐axes from the left/right GaAs strain‐buffer layers; and along the y‐ and *z*‐axes from the top GaAs strain‐buffer layer (Figure 7f). These multiaxial compressive stresses effectively restrict the amplitude of lattice thermal vibrations, thereby stabilizing the band gap against temperature variation in the nanowires. Moreover, the direct coupling between the nanowires and the quantum well in the wire‐well part forms a unified band structure. Therefore, under the action of the nanowires, the thermal shift in the wire‐well part is further restricted. In contrast, the quantum well in the pure‐well part is subjected only to biaxial compressive stresses along the y‐ and *z*‐axes from the upper and lower GaAs layers (Figure 7g). This weaker confinement does not sufficiently suppress lattice thermal vibrations, allowing the emission peak to redshift significantly with increasing temperatures. Thus, the different stress environments explain why the pure‐well emission shifts with temperature while the wire‐well emission remains nearly constant. This mechanism provides a practical route to dual‐wavelength emission with widely tunable wavelength spacing through unified temperature control on a single chip.

To more clearly elucidate this mechanism, the suppression effect of multiaxial stress in nanowires on the band‐gap variation caused by temperature changes is quantitatively evaluated as follows. The emission peak of the pure‐well part redshifts from 963.8 nm at 289 K to 985.7 nm at 321 K, while that of the nanowire‐well part shifts only from 930.6 to 931.8 nm over the same temperature range. The corresponding band‐gap shrinkages are Δ*E*_*g*_^Pure‐well^ = 28.6 meV and Δ*E*_*g*_^Wire‐well^ = 1.7 meV, respectively. Thus, the difference in band‐gap shrinkage between the two parts is Δ*E*_*g*_^Pure‐well^‐Δ*E*_*g*_^Wire‐well^ = 26.9 meV. Accordingly, in the temperature range from 289 to 321 K, it can be reasonably inferred that the presence of nanowires induces an additional band‐gap widening of approximately 26.9 meV in the nanowire‐well part.

According to Vegard's law [58], the strain‐free lattice constant of the nanowires (with an equivalent In content of *x* = 0.16) is given by:

(1) $$a_{\text{Wire}}=x\cdot a_{\text{InAs}}+\left(1-x\right)\cdot a_{\text{GaAs}},$$

where $a_{\text{InAs}}$ and $a_{\text{GaAs}}$ are the lattice constants of InAs and GaAs, respectively. The eigenstrain (*ε*_0_) is defined by the lattice mismatch between the nanowires and the underlying InGaAs quantum well:

(2) $$\varepsilon_{0}=\frac{a_{\text{Well}}-a_{\text{Wire}}}{a_{\text{Wire}}},$$

here $a_{\text{Well}}$ is the lattice constant of the adjacent InGaAs quantum well beneath the nanowires (taken as the equivalent In content of 0.075) also obtained via Vegard's law [58]. Here, the *x*‐direction is defined as the nanowire length direction, the *y*‐direction as the nanowire bottom‐width direction, and the *z*‐direction as the nanowire height direction. Since the nanowire length is much larger than its cross‐sectional dimensions, the strain in the *x*‐direction is taken to be equal to the eigenstrain:

(3) $$\varepsilon_{\text{xx}}=\varepsilon_{0}.$$

For the strains in the y‐ and z‐directions (*ε*_yy_ and *ε*_zz_) of the nanowires, we adopt the Eshelby inclusion theory [59, 60]. The corresponding formulas are

(4) $$\varepsilon_{\text{yy}}=\left({\bar{S}}_{\text{yyyy}}+{\bar{S}}_{\text{yyzz}}\right)\varepsilon_{0}.$$

(5) $$\varepsilon_{\text{zz}}=\left({\bar{S}}_{\text{zzyy}}+{\bar{S}}_{\text{zzzz}}\right)\varepsilon_{0}.$$

To account for the elastic interaction among periodically distributed nanowires, the Mori–Tanaka mean‐field theory is introduced [61]. Then, the strains of the nanowire system in the y‐ and z‐directions can be obtained more accurately as follows:

(6) $$\langle \varepsilon_{\text{yy}}\rangle =\varepsilon_{\text{yy}}^{0}+\frac{1}{3}\varepsilon_{\text{yy}}.$$

(7) $$\langle \varepsilon_{\text{zz}}\rangle =\varepsilon_{\text{zz}}^{0}+\frac{1}{3}\varepsilon_{\text{zz}},$$

here, $\varepsilon_{\text{yy}}^{0}$ and $\varepsilon_{\text{zz}}^{0}$ are the constrained strains of the effective pure‐well part in the y‐ and z‐directions, respectively. $\varepsilon_{\text{yy}}^{0}$ is determined by the lattice mismatch between the pure‐well part (with an equivalent In content of *x* = 0.085) and GaAs:

(8) $$\varepsilon_{\text{yy}}^{0}=\frac{a_{\text{GaAs}}-a_{\text{Well}}}{a_{\text{Well}}}$$

and $\varepsilon_{\text{zz}}^{0}$ is given by the Poisson effect under biaxial strain:

(9) $$\varepsilon_{\text{zz}}^{0}=-\frac{2C_{12}^{\text{Well}}}{C_{11}^{\text{Well}}}\varepsilon_{\text{yy}}^{0},$$

where $C_{11}^{\text{Well}}$ (115.78 GPa) and $C_{12}^{\text{Well}}$ (53.07 GPa) are the elastic constants of InGaAs (with an equivalent In content of *x* = 0.085) obtained by Vegard's law interpolation [58, 62].

The quantities ${\bar{S}}_{\text{yyyy}}$, ${\bar{S}}_{\text{zzzz}}$, ${\bar{S}}_{\text{yyzz}}$, and ${\bar{S}}_{\text{zzyy}}$ are the average self‐ and cross‐response Eshelby tensor components in the y‐ and z‐directions. Since the Eshelby tensor is not constant inside the inclusion but varies with position, evaluating it only at a certain position cannot represent the average strain state over the entire triangular cross section. Therefore, we uniformly sample 1000 discrete points within the triangular cross section, compute the Eshelby tensor at each point, and take the statistical average to obtain more realistic Eshelby tensor components for the nanowire. The Eshelby tensor components computed at a given observation point are given by the following formulas [59]:

(10) $$S_{\text{yyyy}}=-\frac{U}{2}\left[\left(4\lambda +8\mu +4V\lambda +8V\mu \right)\iota_{\text{yy}}+2\lambda \iota_{\text{zz}}-4\lambda \left(\Theta_{\text{yyzz}}+\Theta_{\text{yyyy}}\right)-8\mu \Theta_{\text{yyyy}}\right].$$

(11) $$S_{zzzz}=-\frac{U}{2}\left[\left(4\lambda +8\mu +4V\lambda +8V\mu \right)\iota_{zz}+2\lambda \iota_{yy}-4\lambda \left(\Theta_{zzyy}+\Theta_{zzzz}\right)-8\mu \Theta_{zzzz}\right]$$

(12) $$S_{\text{yyzz}}=-\frac{U}{2}\left[\left(2\lambda +4\mu \right)\iota_{\text{zz}}+\left(4\lambda +4V\lambda \right)\iota_{\text{yy}}-4\lambda \left(\Theta_{\text{yyzz}}+\Theta_{\text{yyyy}}\right)-8\mu \Theta_{\text{yyzz}}\right].$$

(13) $$S_{\text{zzyy}}=-\frac{U}{2}\left[\left(2\lambda +4\mu \right)\iota_{\text{yy}}+\left(4\lambda +4V\lambda \right)\iota_{\text{zz}}-4\lambda \left(\Theta_{\text{zzyy}}+\Theta_{\text{zzzz}}\right)-8\mu \Theta_{\text{zzyy}}\right],$$

here, *μ* is the shear modulus, which, according to the Voigt–Reuss–Hill (VRH) averaging scheme [63], is expressed as

(14) $$\mu =\frac{1}{2}\left[\frac{C_{11}-C_{12}+3C_{44}}{5}+\frac{5\left(C_{11}-C_{12}\right)C_{44}}{4C_{44}+3\left(C_{11}-C_{12}\right)}\right].$$

The elastic constants of the nanowires are C_11_ = 113.12, C_12_ = 52.43, and C_44_ = 56.23 GPa [62]. The parameters U and V are given by

(15) $$U=\frac{1}{8\pi \mu \left(1-\nu \right)}.$$

(16) V=4ν−3.

Poisson's ratio *ν* is determined by the bulk modulus *B* and shear modulus *μ* [63]

(17) $$\nu =\frac{3B-2\mu }{6B+2\mu }.$$

The bulk modulus B is [63]

(18) $$B=\frac{C_{11}+2C_{12}}{3}.$$

The first Lamé constant *λ* is

(19) $$\lambda =\frac{E\nu }{\left(1+\nu \right)\left(1-2\nu \right)},$$

where *E* is Young's modulus [63]

(20) $$E=\frac{9B\mu }{3B+\mu }.$$

The second‐order tensor *ι* and fourth‐order tensor *Θ* are obtained by [59]

(21) $$\iota =\sum_{c=1}^{3}\left(\rho_{c}⊗\Delta \rho_{c}^{⊥}\cdot I_{01}+\Delta \rho_{c}⊗\Delta \rho_{c}^{⊥}\cdot I_{11}\right).$$

(22) $$\Theta =\sum_{c=1}^{3}\left(R_{\rho \rho \rho }^{c}I_{02}+R_{\rho \rho \Delta \rho }^{c}I_{12}+R_{\rho \Delta \rho \Delta \rho }^{c}I_{22}+R_{\Delta \rho \Delta \rho \Delta \rho }^{c}I_{32}\right).$$

Based on the nanowire dimensions, the vertices of the triangular cross section (*P* ^n^) are set as *P* ^1^ (−23.34, 0), *P* ^2^ (66.9, 0), and *P* ^3^ (0, 2.04), For a given observation point O within the triangular cross‐section, we have [59]

(23) $$\rho_{c}=P^{n}-O.$$

(24) $$\Delta \rho_{c}=\rho_{c+1}-\rho_{c}.$$

Note that the ordering of the triangle vertices must be counterclockwise. The symbol $\Delta \rho_{c}^{⊥}$ represents the clockwise rotation of $\Delta \rho_{c}$ by π/2. Additionally [59]

(25) $$I_{01}=\frac{1}{\sqrt{p_{c}u_{c}-q_{c}^{2}}}\left(\arctan\frac{p_{c}+q_{c}}{\sqrt{p_{c}u_{c}-q_{c}^{2}}}-\arctan\frac{q_{c}}{\sqrt{p_{c}u_{c}-q_{c}^{2}}}\right).$$

(26) $$I_{11}=\frac{1}{2p_{c}}\log\frac{p_{c}+2q_{c}+u_{c}}{u_{c}}-\frac{q_{c}}{p_{c}}I_{01}.$$

(27) $$I_{02}=\frac{1}{2\left(p_{c}u_{c}-q_{c}^{2}\right)}\left(\frac{p_{c}+q_{c}}{p_{c}+2q_{c}+u_{c}}-\frac{q_{c}}{u_{c}}+p_{c}I_{01}\right).$$

(28) $$I_{12}=\frac{1}{2\left(q_{c}^{2}-p_{c}u_{c}\right)}\left(\frac{q_{c}+u_{c}}{p_{c}+2q_{c}+u_{c}}-1+q_{c}I_{01}\right).$$

(29) $$I_{22}=\frac{1}{2\left(q_{c}^{2}-p_{c}u_{c}\right)}\left(\frac{q_{c}+u_{c}}{p_{c}+2q_{c}+u_{c}}-u_{c}I_{01}\right).$$

(30) $$I_{32}=\frac{2q_{c}^{2}+q_{c}u_{c}-p_{c}u_{c}}{2p_{c}\left(p_{c}u_{c}-q_{c}^{2}\right)\left(p_{c}+2q_{c}+u_{c}\right)}+\frac{1}{2p_{c}^{2}}\log\left(\frac{p_{c}+2q_{c}+u_{c}}{u_{c}}\right)-\frac{q_{c}\left(3p_{c}u_{c}-2q_{c}^{2}\right)}{2p_{c}^{2}\left(p_{c}u_{c}-q_{c}^{2}\right)}I_{01},$$

where [59]

(31) $$p_{c}=\Delta \rho_{c}\cdot \Delta \rho_{c}.$$

(32) $$q_{c}=\rho_{c}\cdot \Delta \rho_{c}.$$

(33) $$u_{c}=\rho_{c}\cdot \rho_{c},$$

the terms $R_{\rho \rho \rho }^{c}$, $R_{\rho \rho \Delta \rho }^{c}$, $R_{\rho \Delta \rho \Delta \rho }^{c}$, and $R_{\Delta \rho \Delta \rho \Delta \rho }^{c}$ are taken as [59]

(34) $$R_{\rho \rho \rho }^{c}=\rho_{c}⊗\rho_{c}⊗\rho_{c}⊗\Delta \rho_{c}^{⊥}.$$

(35) $$R_{\rho \rho \Delta \rho }^{c}=\rho_{c}⊗\rho_{c}⊗\Delta \rho_{c}⊗\Delta \rho_{c}^{⊥}+\rho_{c}⊗\Delta \rho_{c}⊗\rho_{c}⊗\Delta \rho_{c}^{⊥}+\Delta \rho_{c}⊗\rho_{c}⊗\rho_{c}⊗\Delta \rho_{c}^{⊥}.$$

(36) $$R_{\rho \Delta \rho \Delta \rho }^{c}=\rho_{c}⊗\Delta \rho_{c}⊗\Delta \rho_{c}⊗\Delta \rho_{c}^{⊥}+\Delta \rho_{c}⊗\rho_{c}⊗\Delta \rho_{c}⊗\Delta \rho_{c}^{⊥}+\Delta \rho_{c}⊗\Delta \rho_{c}⊗\rho_{c}⊗\Delta \rho_{c}^{⊥}.$$

(37) $$R_{\Delta \rho \Delta \rho \Delta \rho }^{c}=\Delta \rho_{c}⊗\Delta \rho_{c}⊗\Delta \rho_{c}⊗\Delta \rho_{c}^{⊥}.$$

Thus, neglecting higher‐order terms, the trace of the strain tensor for the nanowires is obtained as

(38) $$Tr\left(\varepsilon \right)=\varepsilon_{\text{xx}}+\langle \varepsilon_{\text{yy}}\rangle +\langle \varepsilon_{\text{zz}}\rangle .$$

For zinc‐blende semiconductors, the band‐gap change Δ*E*_g_ is related to the strain tensor by

(39) $$\Delta E_{g}=a_{h}\cdot Tr\left(\varepsilon \right)+b\left(\langle \varepsilon_{\text{zz}}\rangle -\frac{\varepsilon_{\text{xx}}+\langle \varepsilon_{\text{yy}}\rangle }{2}\right),$$

where $a_{h}$ is the hydrostatic deformation potential and *b* is the shear deformation potential [64, 65].

Thus, the multiaxial compressive stress in the nanowires yields a theoretical band‐gap increase of ∼99.98 meV, which is much larger than the 26.9 meV widening needed from experiments. The theoretical evaluation, while not perfectly matching the experimental data due to the complex multiaxial strain in the nanowires, still clearly indicates that the stress is adequate to compensate for the Varshni thermal band‐gap shrinkage.

At an injection power of 79 mW, as the temperature increases from 289 to 321 K, the integrated PL peak intensities of both the pure‐well part and the wire‐well part monotonically decrease, as shown in Figure 8a. The corresponding integrated‐peak‐intensity ratio of these two parts increases only from ∼1.63 to ∼2.49 across a temperature range of 32 K, as exhibited in Figure 8b. This indicates that as the temperature increases, some carriers migrate from the high‐energy nanowire‐well part to the low‐energy pure‐well part, and the schematic diagram is shown in Figure 8c. However, due to the relatively strong carrier confinement capability of the one‐dimensional nanowires, no large‐scale carrier migration occurs with increasing temperature.

**FIGURE 8:**
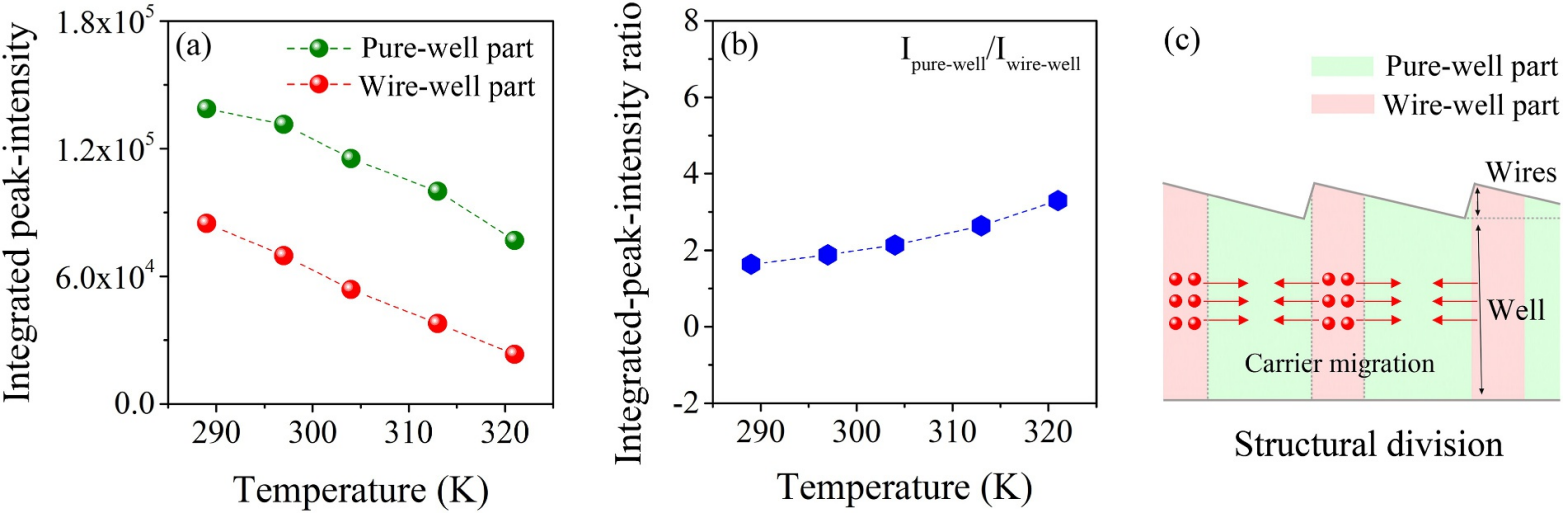
(a) Dynamic integrated peak‐intensities of PL spectra for the pure‐well part and the wire‐well part at various temperatures and an injection power of 79 mW. (b) Integrated‐peak‐intensity ratio of the pure‐well part and the wire‐well part (I_pure‐well_/I_wire‐well_) as a function of temperature at an injection power of 79 mW. (c) Schematic diagram of carriers slightly migrating from the wire‐well part to the pure‐well part as the temperature increases from 289 to 321 K.

### 3.3 Temperature Dependence of the Modal Gain Dual‐Peak Spacing

To characterize the temperature‐dependent variation of their spacing in the modal gain, gain spectra across a range of temperatures and injection powers are examined. The modal gain (*G*) of the nanowire‐well structure is extracted by measuring the amplified spontaneous emission (ASE) spectra from both ends of an edge‐emitting sample and applying the following relationship [66]:

(40) $$G=\frac{1}{L}\ln\frac{\left(1-R_{2}\right)I_{\text{ASE}1}-I_{\text{ASE}2}}{R_{2}\times I_{\text{ASE}2}}.$$

Here, *L* = 1.5 mm is the single‐pass distance of light through the sample. *R*_2_ = 30% represents the reflectivity of the left‐facet of the sample. This end face has not undergone any treatment, and the reflectivity is determined by the material properties. Furthermore, we have carried out finite‐difference time‐domain (FDTD) simulations with Meep to demonstrate the rationality of the chosen reflectivity result. In addition, the anti‐reflection coating is applied to the right‐facet of the sample, and its reflectivity is *R*_1_ ≈ 0. I_ASE1_ and I_ASE2_ denote the ASE intensities detected from the right and the left facets, respectively. Additionally, the schematic diagram of the experimental setup for ASE spectrum measurement is illustrated in Figure 9. The measurement setup is similar to that used for PL spectrum measurement except that the ASE spectra are recorded from both facets of the sample using edge‐emission samples.

**FIGURE 9:**
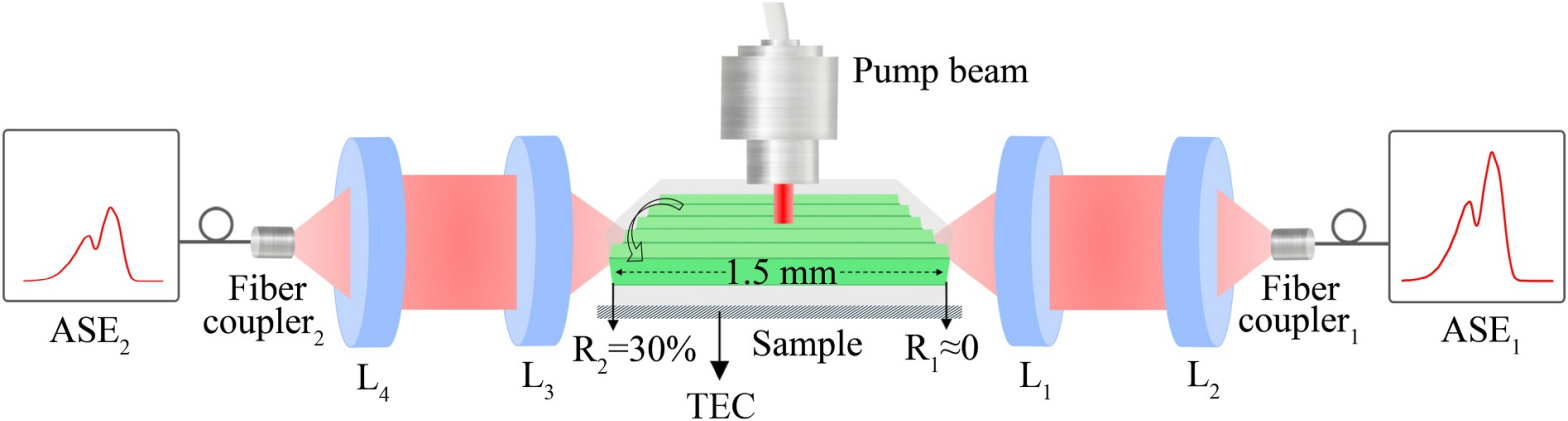
Schematic diagram of the experimental setup for dual‐end ASE (or PL) spectral measurements used in modal gain extraction.

The calculated modal gain spectra across temperatures of 289–321 K at an injection power of 122 mW are illustrated in Figure 10a, which clearly reflect the contributions of the wire‐well part at the short‐wavelength direction and the pure‐well part at the long‐wavelength direction to the total modal gains. To enhance visual clarity due to the relatively small gain differences between different temperatures, the spectra at 313 K, 304 K, 297 K, and 289 K are vertically offset by 5, 10, 15, and 20 cm^−1^, respectively. Under 122 mW injection, the gain peak value variations with temperature of both parts are summarized in Figure 10b. Although the peak gain in each part decreases slightly with rising temperature, the peak gains of the two parts remain comparable under identical conditions (see adjacent columns in Figure 10b), decreasing wavelength selection competition. Furthermore, within the temperature range of 289–321 K, the gain peak of the wire‐well part remains nearly constant at 931.8 nm, whereas the gain peak of the pure‐well part redshifts from ∼965.5 to ∼987.7 nm at 122 mW injection, as exhibited in Figure 10c. This change in trend of the gain curves is consistent with the PL results. Moreover, the temperature‐dependent shift of the gain peaks follows the same variation at higher injection powers (165 and 218 mW) as it does at 122 mW, as shown separately for the wire‐well part (Figure 10d) and the pure‐well part (Figure 10e). Consequently, the spacing between the two gain peaks increases from ∼33.7 to ∼55.9 nm (Δ ≈ 22.2 nm) when the temperature rises from 289 K to 321 K (Figure 10f), corresponding to a tuning rate of approximately 0.69 nm/K. This result is also in good agreement with the PL measurements.

**FIGURE 10:**
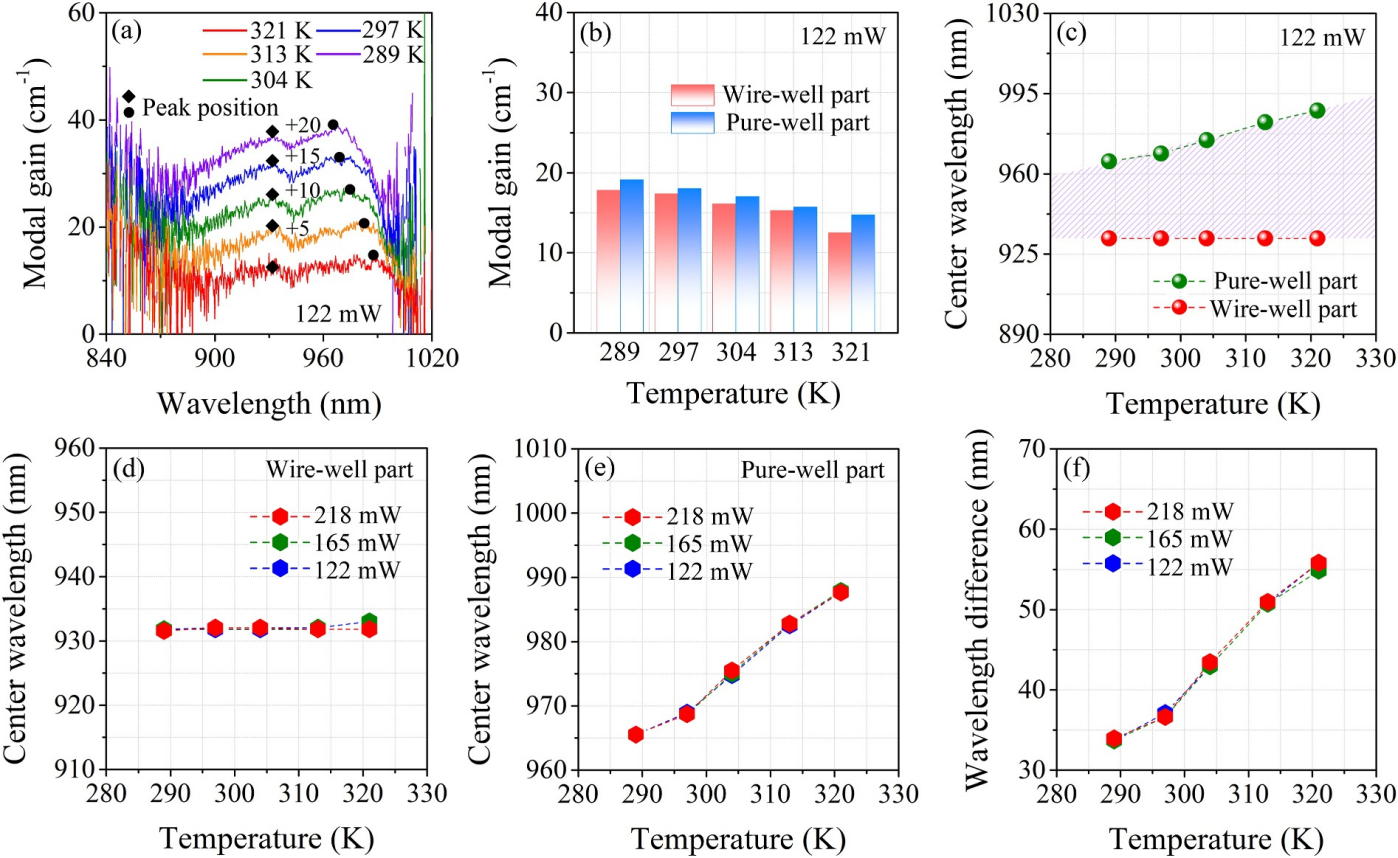
(a) Modal gain spectra of the nanowire‐well sample over the temperature range of 289–321 K at an injection power of 122 mW. (b) Peak modal gain of the wire‐well part and the pure‐well part at different temperatures. (c) Temperature‐dependent peak wavelength shifts for both structural parts at 122 mW injection. Temperature‐dependent peak wavelength variations at different injection powers (122, 165, and 218 mW) for (d) the wire‐well part and (e) the pure‐well part. (f) Dual‐peak wavelength spacing between the two parts versus temperature at different injection powers.

The relatively consistent modal gain peak intensities of the wire‐well and pure‐well parts under identical conditions provide a fundamental advantage for decreasing wavelength selection competition. Crucially, the temperature‐insensitive emission of the wire‐well part, combined with the strongly temperature‐dependent emission of the pure‐well part, creates a unique platform enabling wide, thermally controlled tuning of dual‐wavelength spacing on a single chip. Furthermore, the injection‐power‐independent peak wavelengths ensure controllable tuning of the dual‐wavelength spacing via temperature adjustment. These special optical properties of the nanowire‐well structure offer a straightforward and effective mechanism for the generation of widely tunable dual‐wavelength spacing via unified temperature control. This research and discovery demonstrate significant potential for advancing the development and application of compact tunable dual‐wavelength semiconductor lasers.

## 4 Conclusion

In summary, we propose a novel approach and mechanism for achieving widely tunable dual‐wavelength spacing on a single nanowire‐well chip via simple and unified temperature control based on stress modification in active structures. Driven by the multiatomic step effect, the nanowire‐well composite structure with a laterally periodic distribution of nanowire‐well part and pure‐well part is formed, enabling dual‐wavelength emission. Owing to their distinct stress environments, the two types of parts exhibit markedly different sensitivities to temperature variations, resulting in a widely tunable dual‐wavelength spacing via unified temperature control. Furthermore, the relatively consistent modal gain peak intensities of the wire‐well and pure‐well parts under identical conditions provide a fundamental advantage for decreasing wavelength selection competition. Importantly, the injection‐power‐independent peak positions facilitate stable tuning of the dual‐wavelength spacing via temperature adjustment. This research and discovery provide a promising route and physical mechanism toward compact dual‐wavelength semiconductor lasers with widely tunable wavelength spacing, which holds significant implications for enhancing the dynamic adaptability of laser systems in practical applications.

## Author Contributions

**Yuhong Wang:** conceptualization, investigation, writing – original draft, validation, methodology. **Yiwei Zhang:** data curation, writing – review and editing. **Zihan Jiang:** investigation, writing – review and editing. **Jian Wu:** supervision. **Chunqing Gao:** supervision, validation, resources.

## Conflicts of Interest

The authors declare no conflicts of interest.

## Data Availability

The data that support the findings of this study are available from the corresponding author upon reasonable request.
